# Integrated Approach to Severe Dengue Complicated by Guillain-Barré Syndrome and Multi-organ Failure

**DOI:** 10.7759/cureus.63939

**Published:** 2024-07-06

**Authors:** Madhulika L Mahashabde, Lokesh Kumar

**Affiliations:** 1 General Medicine, Dr. D. Y. Patil Medical College, Hospital and Research Centre, Dr. D. Y. Patil Vidyapeeth, Pune, IND

**Keywords:** guillain-barre syndrome (gbs), covishield vaccine, acute motor axonal neuropathy (aman), intravenous immunoglobulins (ivig), severe dengue fever

## Abstract

This report details the case of a female patient who was admitted with severe dengue, which was further complicated by bilateral pneumonia and multiple organ involvement. The patient also developed quadriparesis, a neurological complication, and had a recent history of vaccination with the COVISHIELD COVID-19 vaccine. A nerve conduction study later determined the condition to be acute motor axonal neuropathy, a variant of Guillain-Barré syndrome (GBS).

While neurological complications, such as GBS, are rare in dengue cases, they can significantly affect patient outcomes. Treatment with intravenous immunoglobulin (IVIG) has proven to be an effective disease-modifying therapy for GBS. IVIG therapy is recognized for its anti-inflammatory and immunomodulatory effects, making it beneficial in certain autoimmune conditions, including those involving the nervous system. However, its use in severe infections or sepsis remains controversial. In this case, IVIG therapy was administered alongside broad-spectrum antibiotics. The patient’s favorable response to IVIG therapy and subsequent clinical improvement highlight the importance of early recognition and targeted intervention for neurological complications in dengue cases.

## Introduction

Dengue, a mosquito-borne viral infection caused by a single-stranded RNA virus belonging to the *Flaviviridae* family (genus *Flavivirus*), poses a significant public health threat in India. The disease is endemic throughout the nation and is primarily transmitted by the *Aedes aegypti* mosquito. Its prevalence is exacerbated by factors such as heavy rainfall, poor sanitation, stagnant water, and insufficient mosquito control measures [1]. Dengue can cause a wide range of symptoms, from moderate flu-like symptoms to severe hemorrhagic fever and even neurological problems. Common signs include fever, body aches, bone pain, muscle pain, and general weakness. In severe cases, hemophagocytic lymphohistiocytosis (HLH) can also occur. According to the data from the National Health Mission (NHM), India reported 193,245 dengue cases and 346 deaths in 2021. In 2022, the number of cases increased to 233,251, while deaths decreased to 303 [2].

The World Health Organization (WHO) classifies dengue cases into categories such as dengue without warning signs, dengue with warning signs, and severe dengue, incorporating severe organ involvement such as liver failure, heart complications, or central nervous system (CNS) manifestations [3]. While neurological complications such as encephalitis, encephalopathy, and meningitis are more commonly noted than Guillain-Barré syndrome (GBS), immunologically mediated issues such as GBS, myelitis, acute disseminated encephalomyelitis (ADEM), and neuropathy represent relatively rare occurrences in dengue cases [4].

The virus comprises four serotypes (DENV1, DENV2, DENV3, and DENV4). Neurological complications are more commonly implicated with DENV2 and DENV3 infections. Immune mechanisms, with molecular mimicry playing a significant role, contribute to the development of delayed neurological complications such as GBS. Despite being rare in dengue cases, GBS warrants attention due to its severity and autoimmune nature. According to a study, neurological manifestations were observed in 2.64% of hospitalized dengue patients, with GBS following dengue virus (DENV) infection being exceptionally rare at only 0.08% [5].

## Case presentation

A 30-year-old female homemaker presented with several complaints. Her chief complaints included acute-onset breathlessness for two days, associated with a continuous high-grade fever with chills and a diffuse, dull-aching headache. She also reported generalized weakness, body aches, and vomiting (four to five episodes per day, non-bilious, non-projectile, without blood staining) for the past week.

The patient received her first dose of the COVISHIELD COVID-19 vaccine one week ago. She reported fever and headache for one day post-vaccination, which resolved with paracetamol. There was no history of any comorbidities or any other significant medical history. Upon physical examination, the patient was conscious and exhibited a clear and coherent orientation to time, place, and person. Examination of vitals revealed a fever with a temperature of 101 °F. Her pulse was regular at a rate of 120 beats per minute. The blood pressure was 80/50 mmHg in the right arm. The respiratory rate was 30 cycles per minute. Oxygen saturation levels were 90% in room air, necessitating the administration of 6 L of oxygen to achieve a saturation of 98%. Further examination unveiled mild pallor. Upon examination of the respiratory system, bilateral coarse crepitations were noted in the chest. Meanwhile, the cardiovascular examination revealed tachycardia with hypotension, and the per-abdomen and CNS examinations were normal.

All routine lab investigations (Tables 1, 2) were performed, including a dengue test, reverse transcription polymerase chain reaction (RT-PCR) for COVID-19, chest X-ray, cultures, and a high-resolution computed tomography (HRCT) of the chest.

**Table 1:** Routine laboratory investigations. ESR: erythrocyte sedimentation rate; CRP: C-reactive protein; LDH: lactate dehydrogenase; SGOT: serum glutamic oxaloacetic transaminase; SGPT: serum glutamic pyruvic transaminase; ALP: alkaline phosphatase

Laboratory Parameters	Results	Laboratory Parameters	Results
Hemoglobin	10.7 g/dL	Total bilirubin	3.72 mg/dL
Total leukocyte count	33,500/µL	Direct bilirubin	2.80 mg/dL
Neutrophils	80%	Indirect bilirubin	0.92 mg/dL
Platelet count	96,000/µL	SGPT	60 U/L
Urea	38 mg/dL	SGOT	138 U/L
Serum creatinine	1.03 mg/dL	ALP	191 IU/L
LDH	351 U/L	D dimer	7894 mg/L
ESR	23 mm/hour	Fibrinogen	218 mg/L
CRP	203 mg/L	Plasma glucose	146 mg/dL

**Table 2:** Dengue test performed using a Rapid Dengue Test kit. The Rapid Dengue Test kit used was manufactured by J. Mitra & Co. (New Delhi, India), with the catalog number IR028050

Dengue Test	Result
Dengue non-structural (NS1) antigen, serum by enzyme immunoassay	Positive
Dengue-IgM antibodies, serum by enzyme immunoassay	Positive
Dengue-IgG antibodies, serum by enzyme immunoassay	Negative

**Figure 1:**
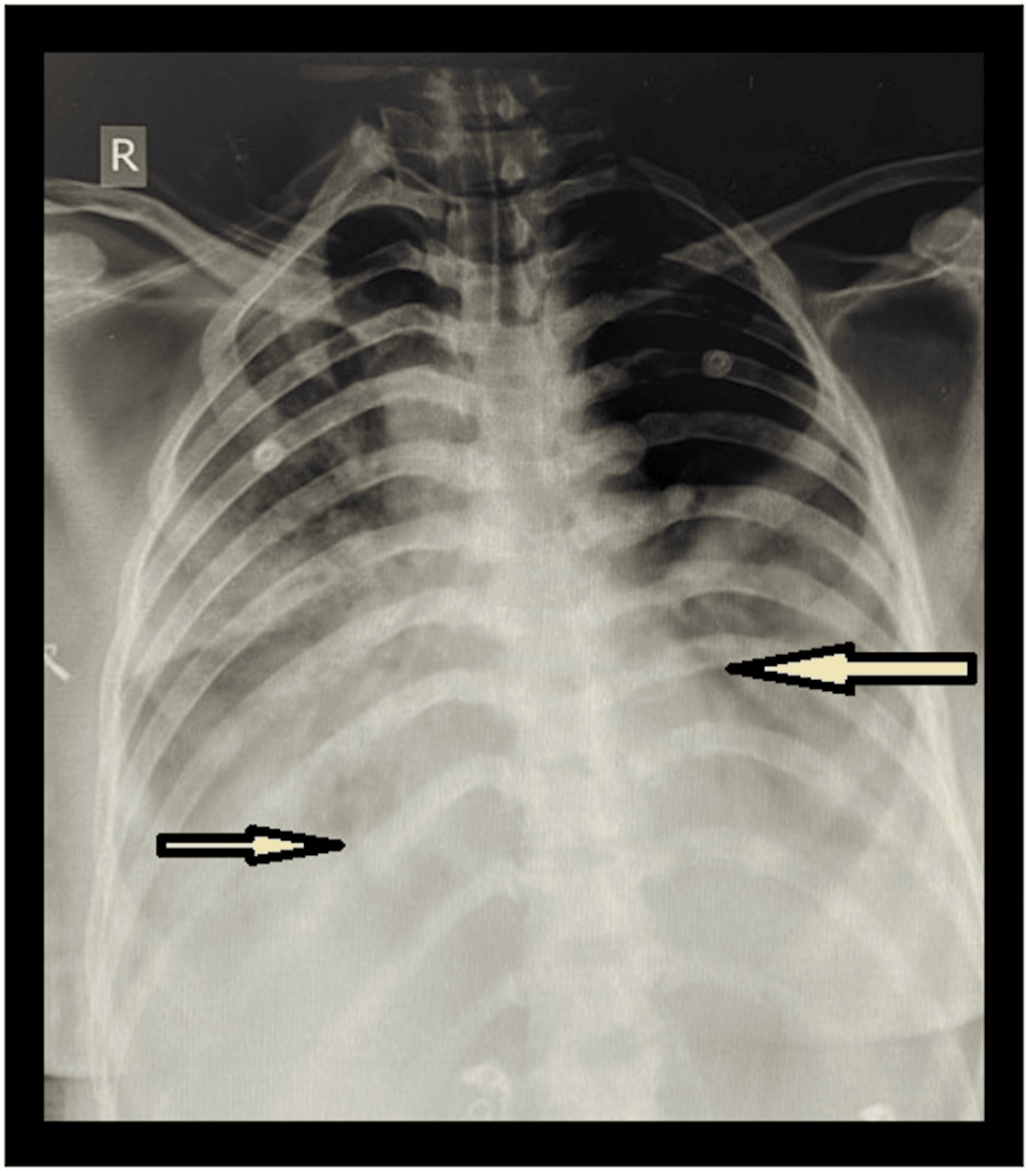
The chest X-ray posteroanterior (PA) view shows non-homogenous opacities with air bronchograms in the bilateral lower zones, suggestive of bilateral lower lobe pneumonia (arrows). The image is original and produced by the authors

The malaria card, Widal test, and COVID RT-PCR were reported as negative. Cultures from blood and urine were sent. The ECG showed sinus tachycardia. The chest X-ray showed bilateral non-homogenous opacities with an air bronchogram suggestive of bilateral lower lobe pneumonia (Figure 1). The echocardiogram was normal. The disseminated intravascular coagulation (DIC) score by the International Society of Thrombosis and Hemostasis (ISTH) was 5, indicative of overt DIC.

**Figure 2:**
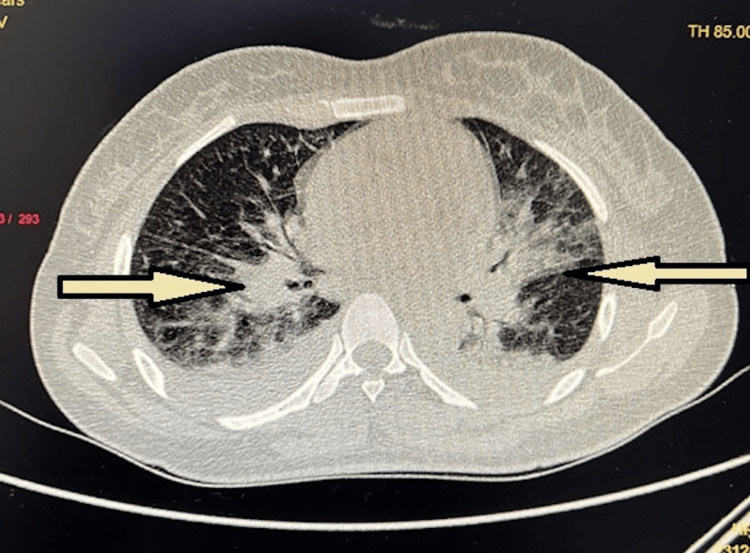
The high-resolution computed tomography (HRCT) scan showed several patches of background consolidation and patchy ground glass opacities (arrows). The image is original and produced by the authors

Based on the evaluation of the patient’s history, clinical examination, and preliminary investigations, the provisional diagnosis was severe dengue with concurrent septic shock and DIC, along with bilateral lower lobe pneumonia. The patient’s critical condition necessitated intensive care unit (ICU) support. Oxygen was administered via a venturi mask at 6 L/minute. A central venous catheter was inserted to monitor central venous pressure. Inotropic support was provided with a continuous noradrenaline infusion. Intravenous antibiotics administered included meropenem (1 g) and tigecycline (50 mg), each given every 12 hours. On the third day of admission, the patient’s respiratory distress worsened, requiring mechanical ventilation with volume control. Sedation with midazolam and neuromuscular blockade with vecuronium were initiated and maintained for 48 hours. An HRCT of the chest revealed background consolidation and patchy ground glass opacities (Figure 2). The findings were categorized as CORADS 5 with a CT severity score of 13/25, indicating extensive lung involvement.

**Figure 3:**
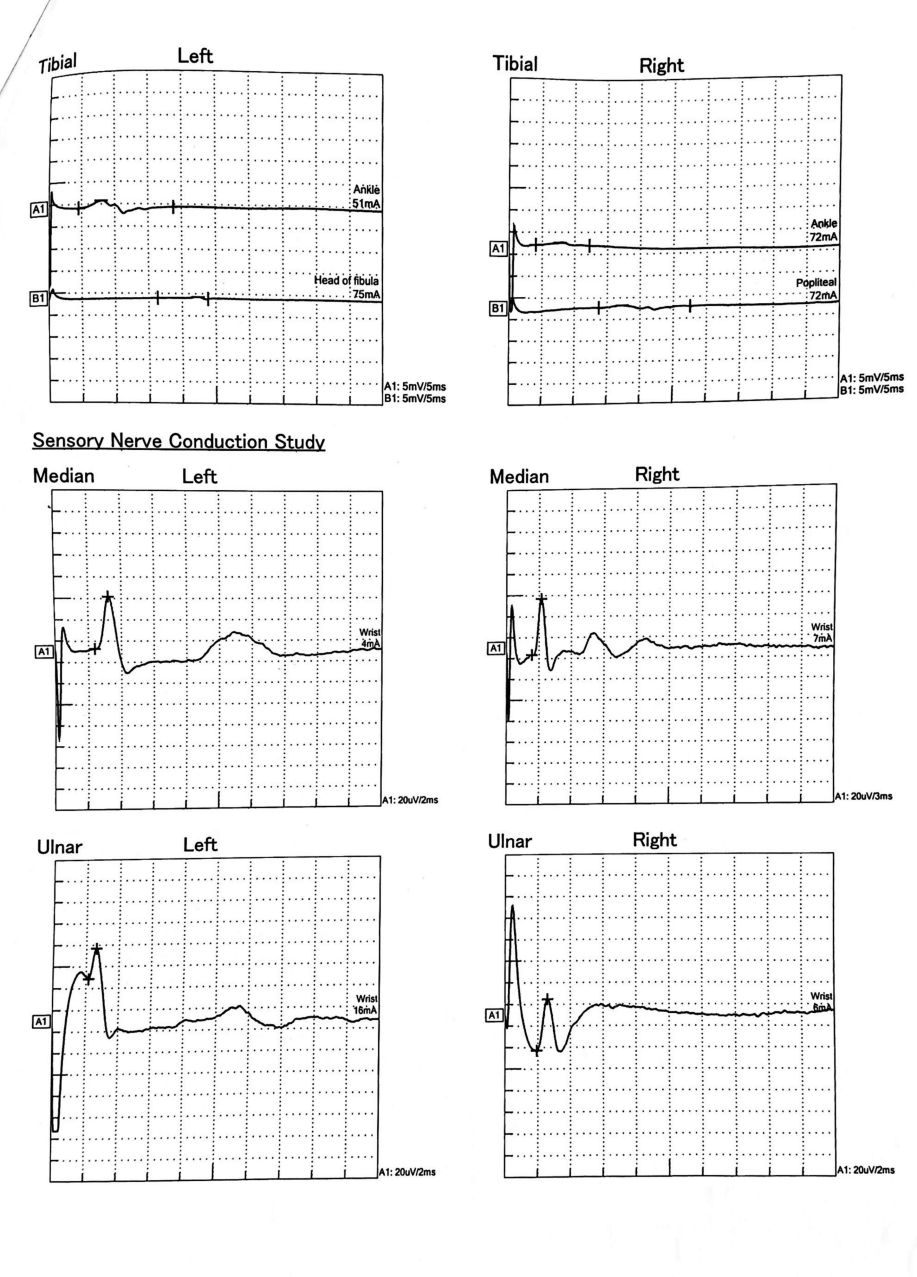
The nerve conduction study (NCS) reveals the absence of F-waves in both tibial nerves.

**Figure 4:**
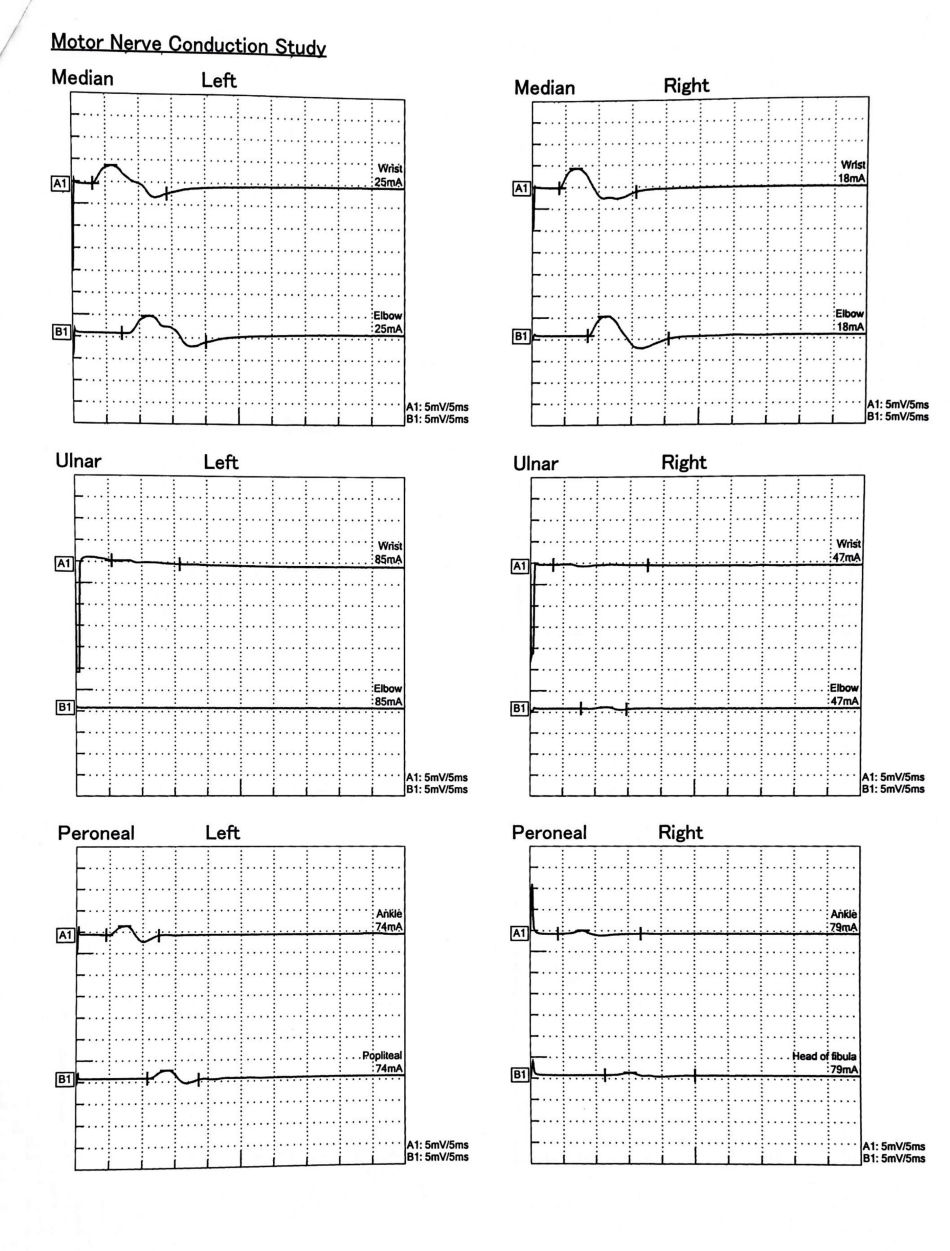
The nerve conduction study (NCS) shows the absence of F-waves in multiple nerves of both the upper and lower limbs.

On the fifth day of admission, sedating and paralyzing agents were discontinued, and the patient exhibited spontaneous eye-opening. However, a new set of neurological symptoms emerged, characterized by weakness in all four limbs with hypotonia. Power was graded as 1/5 in all joints of both the upper and lower limbs. Reflexes were absent, and bilateral plantar responses were mute, which were normal three days earlier. Given these neurological manifestations, a diagnosis of GBS was made, prompting consultation with a neurologist. The diagnosis was further supported by a nerve conduction study, which indicated axonal motor neuropathy with absent F-waves in all tested nerves, suggesting early-stage GBS (Figures 3, 4). The cerebrospinal fluid (CSF) analysis (Table 3) was normal, showing no albuminocytological dissociation.

**Table 3:** The CSF analysis shows the absence of albumin-cytological dissociation. CSF: cerebrospinal fluid; TLC: total leukocyte count

CSF Analysis	Result
Appearance	Clear
Cobweb/coagulum	Absent
Proteins	26 g/dL

The patient’s final diagnosis encompassed a complex, multisystem involvement, including severe dengue with sepsis and shock, bilateral pneumonia, DIC, and GBS. Due to persistent fever spikes and a total leukocyte count of 55,000 with a neutrophil-predominant differential, the endotracheal tube secretions were sent for examination. The culture revealed the presence of *Pseudomonas putida*, which was found to be sensitive to colistin. To target bacterial infections, the following treatments were initiated: intravenous colistin (4.5 MU IV every 12 hours for 12 days), inhaled colistin (1 MU every 12 hours for eight days), levofloxacin (750 mg IV once a day for seven days), and fondaparinux for deep venous thrombosis prophylaxis (2.5 mg once daily). After consultation with a neurologist and obtaining informed consent, the patient received a five-day course of IV immunoglobulin (IVIG) therapy with a total dose of 120 g administered at 2 g/kg body weight. The decision to pursue IVIG therapy was made to address the GBS component of the complex clinical presentation.

The patient demonstrated significant clinical improvement after the IVIG therapy. Motor power in all four limbs improved significantly, progressing from an initial 1/5 to 4/5 in all limbs. Additionally, reflexes showed improvement, advancing to a grade of 2+, indicating a favorable response to treatment.

Despite 14 days of intubation and unsuccessful weaning attempts, a tracheostomy was performed on the 15th day to provide continued ventilatory support. The administration of IV immunoglobulin therapy and colistin, both intravenously and via inhalation, contributed to substantial clinical improvement. The patient’s oxygen requirements decreased over time. On the 38th day post-tracheostomy, notable progress led to the removal of the tracheostomy tube. Chest physiotherapy and spirometry played crucial roles in optimizing respiratory function. Eventually, after 55 days of admission, the patient was discharged, marking a significant milestone in her recovery journey.

## Discussion

Dengue fever is endemic in India, with transmission risk typically higher during and after the monsoon season due to increased mosquito activity. Initially believed to be non-neurotropic, PCR testing has shown the presence of the DENV in CSF, providing evidence of the virus’s neuroinvasion. Furthermore, in encephalitis cases, DENV4 has been found in brain cells and CSF [6]. DENV, a neurotropic agent, causes coagulopathy and plasma leakage by directly invading the brain and enhancing its effects through antibodies [7]. Neurological manifestations have been documented globally across various age groups. Contributing factors include elevated body temperature, increased hematocrit levels, thrombocytopenia, skin rash, and liver dysfunction. CNS involvement is linked to virus-induced systemic disruption, ultimately precipitating encephalopathy.

GBS is a rapidly progressing autoimmune polyradiculoneuropathy, typically presenting with bilateral symmetric motor paralysis and reduced tendon reflexes, sometimes including sensory deficits. While rarely associated with DENV, GBS is frequently linked to various viruses, including cytomegalovirus, Epstein-Barr virus, *Mycoplasma pneumoniae*, varicella-zoster virus, herpes simplex virus, hepatitis A, B, and C viruses, influenza virus, HIV, and bacterial infections such as *Campylobacter jejuni*, *Haemophilus influenzae*, and *Escherichia coli*. Over 85% of GBS patients fully recover functionally within a few months to a year. Under ideal circumstances, the mortality rate is less than 5%, usually due to subsequent pulmonary complications [8]. Dengue fever has been linked to GBS, and given the patient’s recent COVISHIELD COVID-19 vaccination, its potential role in GBS cannot be disregarded. Some documented cases of GBS following the COVISHIELD vaccination have occurred, typically in the second week post-vaccination [9].

## Conclusions

Prompt initiation of IVIG therapy is crucial in treating GBS. It is imperative to maintain a heightened degree of clinical suspicion regarding neurological problems to facilitate prompt diagnosis and early initiation of treatment. This case underscores the need for increased vigilance, particularly in areas where dengue is common, and illustrates our growing understanding of dengue’s neurological effects. The patient’s favorable outcome after 55 days of hospitalization highlights the importance of early recognition and intervention. It also underscores the significance of a comprehensive and adaptive medical approach, reflecting the healthcare team’s collaborative efforts in addressing such complex and multifaceted cases.
